# Supplementary material for: A graphical exploration of the relationship between parasite aggregation indices
Source: PLoS One. 2024 Dec 31;19(12):e0315756. doi: 10.1371/journal.pone.0315756 (PMC11687863; doi:10.1371/journal.pone.0315756)
Supplement: S1 File — (PDF) [file pone.0315756.s001.pdf]

SUPPLEMENTARY MATERIAL: A GRAPHICAL EXPLORATION OF THE RELATIONSHIP BETWEEN PARASITE AGGREGATION INDICES

R. McVINISH and R.J.G. LESTER
School of Mathematics and Physics, University of Queensland
School of Biological Sciences, University of Queensland

S1. Hoover index of the negative binomial distribution

In parasitology the negative binomial distribution is usually parameterised in terms of the mean m and k. The probability mass function is then

$$f(x;k,m) = \binom{k+x-1}{k-1}\left(\frac{k}{k+m}\right)^k\left(\frac{m}{k+m}\right)^x, \quad x\in\mathbb{N}_0,$$

and we write $\mathsf{NB}(k,m)$. Let $F(\cdot;k,m)$ denote the cumulative distribution function of the $\mathsf{NB}(k,m)$ distribution. The first moment distribution of the $\mathsf{NB}(k,m)$ distribution, $F^{(1)}(\cdot;k,m)$, is

$$F^{(1)}(x;k,m) = \frac{\sum_{y\le x} y\, f(y;k,m)}{m}.$$

For any non-negative integer x

$$\begin{aligned}
\frac{x\,f(x)}{m} &= \frac{x}{m}\binom{k+x-1}{k-1}\left(\frac{k}{k+m}\right)^k\left(\frac{m}{k+m}\right)^x\\
&= \frac{x}{m}\frac{(k+x-1)!}{(k-1)!x!}\left(\frac{k}{k+m}\right)^k\left(\frac{m}{k+m}\right)^x\\
&= \frac{(k+x-1)!}{k!(x-1)!}\left(\frac{k}{k+m}\right)^{k+1}\left(\frac{m}{k+m}\right)^{x-1}\\
&= \frac{(k+x-1)!}{k!(x-1)!}\left(\frac{k(1+1/k)}{(k+m)(1+1/k)}\right)^{k+1}\left(\frac{m(1+1/k)}{(k+m)(1+1/k)}\right)^{x-1}\\
&= \binom{(k+1)+(x-1)+1}{(k+1)-1}\left(\frac{k+1}{k+1+(m+m/k)}\right)^{k+1}\left(\frac{m+m/k}{k+1+(m+m/k)}\right)^{x-1},
\end{aligned}$$

which is the probability mass function of the $\mathsf{NB}(k+1, m+m/k)$ distribution evaluated at $x-1$. Hence,

$$F^{(1)}(x;k,m) = F(x-1;k+1,m+m/k).$$

Arnold and Sarabia (2018, Lemma 5.3.3) states that the Hoover index can be expressed as

$$H = F(m;k,m) - F^{(1)}(m;k,m).$$

Hence,

$$H = F(m;k,m) - F(m-1;k+1,m+m/k).$$

S2. Lorenz ordering of the negative binomial distribution

Following Gastwirth (1971), the Lorenz curve of a distribution with distribution function F is given by

$$L(u) = \frac{\int_0^u F^{-1}(y)\,dy}{\int_0^1 F^{-1}(y)\,dy} = \frac{\int_0^u F^{-1}(y)\,dy}{\mu}, \quad u \in [0,1],$$

where $F^{-1}(y) = \sup\{x : F(x) \le y\}$ for $y \in (0,1)$ and $F^{-1}(y) = \sup\{x : F(x) < 1\}$ for $y = 1$. The Lorenz curve defines a partial order on the class of all distributions on $[0,\infty)$ with finite mean (Arnold and Sarabia, 2018, Definition 3.2.1).

Definition: For random variables X and Y with respective Lorenz curves denoted L_X and L_Y, X is less than or equal to Y in the Lorenz order, denoted $X \le_L Y$, if $L_X(u) \ge L_Y(u)$ for every $u \in [0,1]$.

Closely related to the Lorenz order is the convex order (Shaked and Shanthikumar, 2007, subsection 3.A.1).

Definition: For random variables X and Y such that $\mathbb{E}\,\phi(X) \le \mathbb{E}\,\phi(Y)$ for all convex functions $\phi : \mathbb{R} \to \mathbb{R}$ for which the expectations exist. Then we say that X is smaller than Y in the convex order, denoted $X \le_{\mathrm{cx}} Y$

The convex order relates to the Lorenz order in the sense that

$$\frac{X}{\mathbb{E}X} \le_{\mathrm{cx}} \frac{Y}{\mathbb{E}Y}$$

if and only if $X \le_L Y$, provided the expectations exist (Shaked and Shanthikumar, 2007, equation 3.A.33) or (Arnold and Sarabia, 2018, Corollary 3.2.1).

Theorem 1. *(a) For any $k > 0$ and $0 < \mu_1 < \mu_2$, $\mathsf{NB}(k,\mu_2) \le_L \mathsf{NB}(k,\mu_1)$.*
(b) For any $\mu > 0$ and $0 < k_1 < k_2$, $\mathsf{NB}(k_2,\mu) \le_L \mathsf{NB}(k_1,\mu)$.

Proof. For part (a), let $X_2 \sim \mathsf{NB}(k,\mu_2)$. Conditional on X_2, let $X_1 \sim \mathsf{Binomial}(X_2, \mu_1/\mu_2)$. Then $X_1 \sim \mathsf{NB}(k,\mu_1)$. As $\mathbb{E}(X_1|X_2) = (\mu_1/\mu_2)X_2$ and $\mathbb{E}X_1 = (\mu_1/\mu_2)\,\mathbb{E}X_2$, (Arnold and Sarabia, 2018, Theorem 3.4) implies $(\mu_1/\mu_2)\,X_2 \le_L X_1$. Since the Lorenz order is invariant under a change of scale, $\mathsf{NB}(k,\mu_2) \le_L \mathsf{NB}(k,\mu_1)$.

For part (b), standard conditioning arguments show that if $(N,\ t \ge 0)$ is a standard Poisson process and $\Lambda \sim \mathsf{Gamma}(\alpha,\beta)$ (Gamma distribution with shape parameter α and rate parameter β), then $N_\Lambda \sim \mathsf{NB}(\alpha, \alpha/\beta)$. Let $T_i \sim \mathsf{Gamma}(k_i, k_i/\mu)$.

It is known that for every convex function ϕ, $\mathbb{E}\phi(N_t)$ is a convex in t (Schweder, 1982, Proposition 2). If we can show that $T_1 \le_{cx} T_2$, then the result will follow from Shaked and Shanthikumar (2007, Theorem 3.A.21).

By construction $\mathbb{E}T_1 = \mathbb{E}T_2$. Let g_i be the probability density function of $\mathsf{Gamma}(k_i,\beta_i)$. Then $T_1 \le_{cx} T_2$ if $g_2 - g_1$ exhibits exactly two sign changes in the sequence +, -, + (Shaked and Shanthikumar, 2007, Theorem 3.A.44). As the log function is increasing,

$\log g_2 - \log g_1$ has the same sequence of sign changes as $g_2 - g_1$. Then

$$\begin{aligned}&\log g_2(x) - \log g_1(x)\\&= (k_2-1)\log x - \frac{k_2}{\mu}x - \left((k_1-1)\log x - \frac{k_1}{\mu}x\right) + C\\&= (k_2-k_1)\log x - \frac{(k_1-k_2)}{\mu}x + C,\end{aligned}$$

where C depends on k_1, k_2 and μ but not x. There must be at least one sign change since both g_1 and g_2 integrate to one. For $k_2 > k_1$ this function is concave so there must be two sign changes. As this function is positive for $x \to 0$ and $x \to \infty$ when $k_2 > k_1$ we have shown $T_1 \leq_{cx} T_2$. This completes the proof. □

S3. Stability of the Gini index

Contour plots of the derivative of Gini index illustrating the stability of the Gini index with respect to m and k in the region of the empirical data (dots). The derivative with respect to m remains small over a wide range of pairs (m, k). The derivative with respect to k tends to be large in absolute value, particularly for pairs (m, k) corresponding to the empirical data.

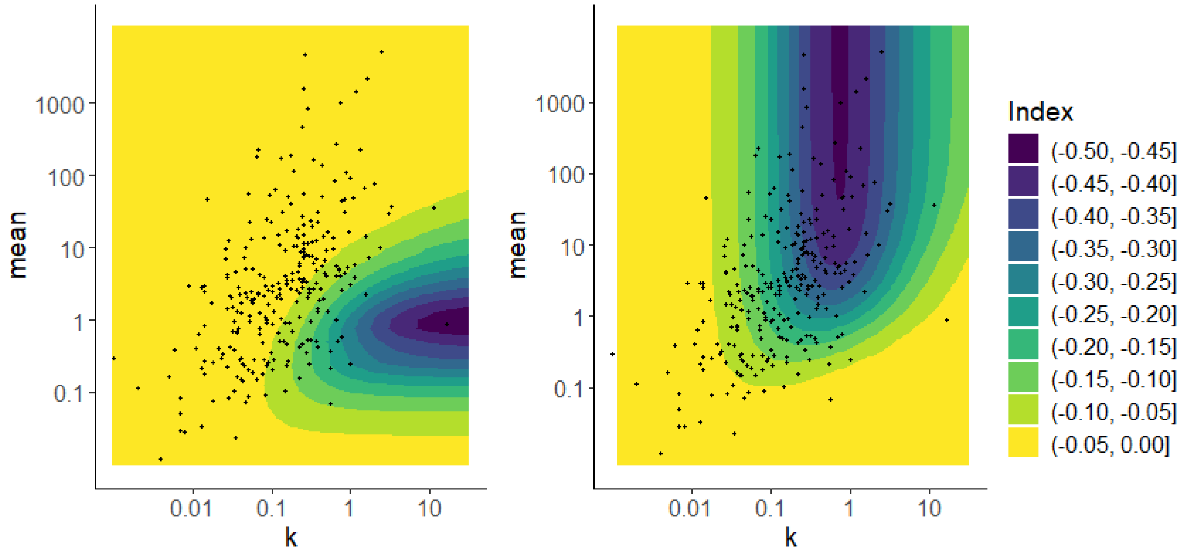

Figure 1. Figure: (Left) Contour plot of the derivative of the Gini index with respect to m. The large yellow area shows the index does not change quickly as m increases. (Right) Contour plot of the derivative of the Gini index with respect to k. Many of the values from Shaw and Dobson fall in the region of faster change with respect to changes in k.

References

B.C. Arnold and J.M. Sarabia. *Majorization and the Lorenz order with applications in applied mathematics and economics.* Springer, New York, 2018.

J.L. Gastwirth. A general definition of the lorenz curve. *Econometrica*, 39:1037–1039, 1971.

T. Schweder. On the dispersion of mixtures. *Scandinavian Journal of Statistics*, 9:165–169, 1982.

M. Shaked and J.G. Shanthikumar. *Stochastic orders*. Springer, New York, 2007.
