# Supplementary material for: A graphical exploration of the relationship between parasite aggregation indices
Source: PLoS One. 2024 Dec 31;19(12):e0315756. doi: 10.1371/journal.pone.0315756 (PMC11687863; doi:10.1371/journal.pone.0315756)
Supplement: Attachment — Submitted filename: Response to Reviewers.docx [file pone.0315756.s002.docx]

**Response to Reviewers of ‘A graphical exploration. . .’**

*Reviewer #1* and *Reviewer #2*	No critical comments.

*Reviewer #3*

1. Introduction
   - The introduction has been expanded in lines 32-42.
   - Further justification for contour plots given in lines 253-258.
   - References 1 to 5 have been added to the Introduction (lines 36 - 42).
2. Methods
   - More details on how the plots were constructed in lines 85-87. We assume the reviewer did not want an explanation of the working of the contouring function in ggplot2.
   - A pair of prevalence and 𝑚 is consistent with the negative binomial distribution if and only if the equation in Line 95 has a solution. See lines 92-99.
   - Justification for log scaling given in lines 89-90.
3. Results
   - Mixing results and interpretation is common in mathematical biology papers. We tried to separate them, but we feel the present approach is clearer.
   - ‘Relationship between 𝑚 and 𝑘’ has been reworded to clarify (lines 119-121)
   - We have rephrased some of the discussion concerning the stability of the indices. Contour plots of the derivative of Gini index are provided in Supplementary data 2 (line 164).
4. Discussion
   - Preferences of Hoover over Gini, example given (lines 239-241).
5. Figures
   - Concerning the colour palette, we used the viridis colour palette in the contour plots. The palette is designed to be easier to read by those with colour-blindness, and print well in grey scale. See, Garnier, et al, (2024). viridis(Lite) - Colorblind-Friendly Color Maps for R. doi:10.5281/zenodo.4679423, viridis package version 0.6.5, <https://sjmgarnier.github.io/viridis/>.
   - Concerning labels for the contour lines, there is already a lot of detail in the figures. We think adding labels to the contour lines will make the graphs less readable.
   - The font size has been increased for axis labels and legend.

Minor comments.

1. Poulin’s D is now explained in lines 52-54.
2. Hoover and Gini indices now named in lines 52 – 55.
3. ‘Reported prevalence values’ now added, line 135.
4. ‘Bumps that occur’ now more fully explained, lines 169-172.
5. ‘decreases in the Lorenz order’ addressed in lines 215-216.
6. ‘unravel other complex relationships’. The Discussion has been rewritten and this phrase removed.

Questions for Authors

1. The results may be substantially different if the distribution is very different to the negative binomial distribution. This has been addressed in lines 260-267.
2. We explain in lines 252 – 258 that when the relationship is nonlinear (as is the case for these indices), correlation is misleading.
3. The potential application of our insights is discussed in the penultimate paragraph, lines 300-305.
4. The difference between Gini and Hoover is addressed in lines 237-241.
5. In relation to previous work on relationships between aggregation indices in parasitology, previous work is sparse. We discuss the conclusions of a recent large paper on this topic by Merrill et al., at length, lines 269 – 281. Indeed, it was this publication that stimulated our paper (line 60).

Reviewers #4, 5 and 6. No critical comments.
